# The universal health coverage crossroads for Mauritius, a small island developing state: progress, paradox, and the path forward

**DOI:** 10.3389/fpubh.2026.1819440

**Published:** 2026-04-17

**Authors:** Ajoy Nundoochan

**Affiliations:** WHO Country Office, Port Louis, Mauritius

**Keywords:** aging population, catastrophic health expenditure, efficiency, equity, universal health coverage

## 1 Introduction

A patient arrives at the emergency unit at 1 p.m. with chest pain. The triage nurse immediately checks the vital signs, and treatment begins without any financial procedures. A specialist is called, diagnostic tests are performed, and the patient is admitted for observation. By morning, the patient is deemed fit for discharge, given medications, follow-up appointments, and care instructions. The entire interaction carries no price tag at the point of care.

This is the case every day, in Mauritius, a small island developing state, where out of a population of 1.24 million, 14,000 of patients who walk into public hospitals and health centers without paying a single rupee. Healthcare in Mauritius is treated as a fundamental right rather than a commodity—a principle anchored not only in rhetoric but translated into concrete daily action. This free healthcare system—sustained for over four decades—is the cornerstone of the Mauritius welfare state. It is a matter of national pride for everyone, regardless of income, are able to access medical care whenever and wherever they need it. Beyond routine care, the state also provides financial assistance—capped at a fixed amount—for patients requiring surgeries that can only be performed abroad.

Yet, Mauritius today stands at a crossroad to sustain Universal Health Coverage (UHC)—a key target of the Sustainable Development Goals. The evidence tells a story of genuine achievement, but emerging trends, if left unaddressed, could put at risk what has been so carefully built. This article examines that story across three perspectives: the progress achieved, the paradoxes that undermine it, and the path forward that evidence and equity demand. Health system value is assessed along two core dimensions: equity (“value for many”) and efficiency (“value for money”)—alongside the long-term financial sustainability of the system (1).

## 2 A transformative policy choice: building a welfare state to bolster universal health coverage

The bold decision to make healthcare free has yielded compounding dividends. Once described as a country with gloomy economic prospects in the 1960's, Mauritius has undergone a remarkable transformation—ascending to upper-middle-income status within four decades. The investment in human capital was foundational, not incidental to this rise. A healthy, educated population provided the productive base upon which sustained growth was built—a legacy to be maintained at all cost.

The results on the health coverage front are measurable. The UHC Service Coverage Index rose from 43 in 2000 to 66 in 2021. The latest draft WHO/World Bank UHC Monitoring Report (2025) estimates a leapfrog to 75—placing Mauritius second in Africa and only thirteen index points behind highly performing countries (2). For a small island developing state with limited resources, this is a genuinely exceptional achievement.

A decisively indicative test of any universal health system is not simply whether services are free, but whether they reach the most vulnerable as well. On this measure, Mauritius performs impressively. A 2021 study reveals a strongly pro-poor pattern: the poorest 20% of Mauritians receive 19.25% of total public health benefits, while the wealthiest 20% receive only 8.29%. For outpatient care at public facilities, the poorest quintile alone accounts for 45% of all benefits; for inpatient admissions, the two lowest quintiles combined capture 49%. The concentration index—a statistical measure where negative numbers indicate pro-poor distribution—confirms this: outpatient services score −0.33 (strongly pro-poor), inpatient services −0.14, and the overall public sector −0.26 (3). Public health spending is flowing effectively, as it should, to those who need it most. Critically, this pro-poor distribution is not merely a statistical artifact—it reflects deliberate policy choices blended with geographically dispersed network of health facilities, zero user fees at every public facility, and the active removal of financial barriers that in many comparable countries continue to deter the poorest from seeking care.

### 2.1 The paradox beneath the surface

Beneath this generous façade, three interconnected paradoxes undermine the equity and efficiency achievements of the public system: the rise of catastrophic health expenditure notwithstanding free care, significant hospital inefficiencies, and the looming fiscal pressures of a rapidly aging population.

### 2.2 Catastrophic health expenditure challenging the welfare state system

Following the flow of health expenditure reveals a striking duality. While the public sector distributes benefits in a strongly pro-poor manner, the private healthcare sector exhibits the opposite pattern. The concentration index for private care stands at +0.27—firmly pro-rich. The wealthiest quintile captures 41.90% of private sector benefits, while the two poorest quintiles combined receive only 23.86%. When both sectors are considered together, the overall equity gains of the public system are substantially offset by the expanding and deeply inequitable private sector (3).

At the household level, out-of-pocket spending is rising. Catastrophic health expenditure—defined as out-of-pocket health expenditure exceeding 10% of household income—affected 6.52% of Mauritian households in 2006, rising to 8.7% by 2017 (4). The latest WHO/World Bank UHC Monitoring Report (2025) estimates that by 2023, 12% of Mauritian households face financial hardship defined as out-of-pocket health expenditure exceeding 40% of household discretionary budget (2). This is deeply troubling in a country that prides itself on universal, free healthcare. Why are economically vulnerable households—precisely those free public care was designed to protect—incurring catastrophic costs in the private sector? The scale and trend point to systemic gaps: insufficient trust in public services, perceived quality deficits, and a system that does not always meet patients where they are.

Drug expenditure adds another layer of concern. The latest National Health Accounts estimates that MUR 4.67 billion is spent annually on medicines—MUR 3,750 per person (5). Private-sector pharmaceutical prices are dramatically inflated: in 2019 innovator brand medicines costed 10.25 times international reference prices, while even the lowest-priced generics costed 4.87 times the reference price (6). These pricing disparities are a principal driver of catastrophic health expenditure, pushing vulnerable households into financial hardship.

### 2.3 Hospital inefficiency as a bottleneck to coverage

Public hospitals absorb approximately 70% of government general health expenditure (GGHE). With limited fiscal space, every cent lost to inefficiency is a cent diverted from primary care, household financial protection, and better population health outcomes. A Stochastic Frontier Analysis in 2020 revealed that public regional hospitals in Mauritius operate at only 83%−89% of their potential efficiency. Improving technical efficiency to 95% is projected to generate savings of MUR 880 million (4.8% of GGHE) by 2025–2026; achieving full efficiency would yield MUR 1,614 million (8.6% of GGHE)—transformative resources that could expand health care coverage and reduce out-of-pocket burdens (7).

Hospital inefficiency is deeply linked to weak primary care utilization. It is estimated that at least 50% of hospital emergency cases could have been appropriately managed at primary health care (PHC) facilities (7). The result is a self-reinforcing dysfunction: hospitals overloaded with routine cases, PHC centers underused despite prior investments, and capacity for genuinely complex acute care compressed. Root causes arguably include inadequate consultation times, insufficient preventive services for pre-diabetic and hypertensive populations, and deeply ingrained patient perceptions that hospital care is inherently superior, even for minor ailments.

Non-communicable diseases account for nearly 90% of total burden of diseases. The prevalence of diabetes (over 45% of the adult population is either diabetic or prediabetic) hypertension, and obesity are among the highest in the world. Still, preventive care remains chronically underused. Patients underestimate the value of routine screenings, lifestyle programmes, and medication adherence. In many cases this complacency leads to deteriorating conditions beyond the point of easy management. What could have been handled in a primary health care clinic ends up in hospital admission. This pattern of avoidable, late-stage demand congests public facilities, strains clinical resources, and crowds out care for those with acute needs—implying underuse of prevention at one end of the system directly generates overload at the other.

### 2.4 A demographic ticking time bomb

Mauritius has rapidly transitioned from a young to an aging society. Fertility rates have fallen from six children per woman in the 1960s to 1.4 by 2020, while life expectancy has risen to 76.4 years. The share of people aged 65 and above currently stands at 13%, projected to rise to 22% by 2040 and 29% by 2060. Among those already aged 60 and older, 24.5% report difficulties performing daily activities due to health issues—translating directly into heightened, sustained demand for healthcare and long-term care services (8, 9).

The Population Aging Financial Sustainability for Health Systems (PASH) Simulator projects that age-related costs will require an additional US$67 per person annually by 2060 (0.7% of GDP), rising to US$127 per person (1.3% of GDP) under pessimistic scenarios. As the working-age population contracts, tax revenues will decline while rising pension and social security obligations crowd out public funds available for healthcare—creating an intensifying fiscal gap the system was not designed to absorb (8, 9).

## 3 Discussion

### 3.1 Path forward: evolution in service of the welfare state

The challenges identified are not insurmountable, on the contrary the evidence maps a concrete, actionable agenda across three mutually reinforcing fronts.

#### 3.1.1 Restore the gatekeeping function of primary healthcare

The most immediate efficiency gain lies in restoring primary care to its required functions. If 50% of hospital cases seen which could well have been managed at the primary health care facilities, the system must create enabling conditions—through longer consultation times, stronger preventive services for non-communicable diseases (especially diabetes and hypertension), and sustained public education—that make primary care the default first point of contact.

This is not merely an efficient measure; it is the foundation of any responsive, patient-centered system that builds public trust and reduces the financial pressures driving households toward costly private alternatives. Evidence-based frameworks such as the WHO's Performance Assessment Tool for Quality Improvement in Hospitals (PATH) and the Service Availability and Readiness Assessment (SARA) provide ready-made tools. Their systematic application across public hospitals, combined with meaningful performance incentives, could unlock between MUR 880 million and MUR 1,614 million in fiscal space to reinvest in equity, quality, and sustainability (7).

Ensuring that there is adequate workforce capacity at the PHC level is critical. Otherwise redirecting half of hospital emergency cases to PHC centers will defeat the very objective of gatekeeping reform. Ultimately creating bottlenecks rather than efficiency gains. Coupled with this, the digitalization of the main hospitals needs to be accelerated to the primary health care facilities. Only then referral tracking and outcome monitoring across care levels would be achievable.

An important trade-off to be mindful is that patients have accessed hospitals as a first point of contact for over four decades. Restricting this access may risk eroding the long-standing public trust and driving unintentionally people toward private care. The transition needs to be carefully planned as it may entail service disruption and public resistance before the benefits of improved care can be reaped.

An inherent systemic risk that needs to be flagged, is that PHC reforms implemented without visible quality improvements may entrench the very perception they seek to dismantle—that hospitals provide superior care—further increasing avoidable hospital use and private sector reliance. In some cases, worsening financial protection and the incidence of catastrophic payments in low-income households.

#### 3.1.2 Strengthen the pharmaceutical ecosystem

Addressing inflated medicine prices requires expanding the domestic production base and reforming pharmaceutical distribution and retail structures. The local market, however, is too small to sustain this ambition, a significant share of output would need to be exported. The African Union's Pharmaceutical Manufacturing Plan for Africa and the African Medicines Agency Treaty provide a regional architecture increasingly prioritizing local production as a pillar of health sovereignty. Mauritius fits well in this architecture, with strong rule of law, financial services infrastructure, and established trade links. Mauritius has an emerging strategic opportunity: to position itself as a pharmaceutical manufacturing hub for Africa, a continent with a market for pharmaceuticals valued at an estimated US$ 14 billion in 2019, with imports accounting for 70%−90% of its medicines consumed (10). Realizing this opportunity requires attaining WHO Global Benchmarking Tool (GBT) maturity level 3, the minimum standard for a credible national medicines regulatory authority. Achieving this standard would strengthen the domestic regulatory environment, enhance investor confidence, and help address the inflated private-sector drug prices that are currently driving catastrophic health expenditure among vulnerable households. However, attaining the WHO GBT Level 3 maturity is a multi-year undertaking, requiring an independent national regulatory authority and sustained institutional capacity building.

Mitigating the high costs of medicine in the private sector—reaching up to 10.25 times international reference prices in 2019—will require challenging entrenched commercial interests across the pharmaceutical distribution and retail sector. However, the risk that private actors will resist pharmaceutical reform cannot be overlooked.

#### 3.1.3 Plan foresightedly for aged care

The demographic transition is already underway; the only choice is whether adaptation is managed ahead of the curve or under fiscal duress. A developed geriatric care workforce at scale is lacking. Building this capacity requires immediate investment in curriculum development and training pathways—but these cannot be achieved quickly. In the same vein, adequate long-term care infrastructure in both community and residential settings is limited. Any seamless transition from acute, hospital-based care to community and residential long-term care will require physical infrastructure and coordinated care systems, all of which are currently under development.

A multi-pronged strategy is needed: investing now in geriatric care infrastructure and workforce capacity; scaling preventive and chronic disease management programmes to compress morbidity in later life; and developing long-term care frameworks that reduce acute hospital dependency among older Mauritians. Within PHC, care pathways that screen for and prevent declines in intrinsic capacity including loss of mobility, malnutrition, visual impairment, hearing loss, cognitive decline, and depression—are essential to managing the health consequences of demographic aging.

### 3.2 Limitations

Only a selective body of evidence has been considered, and the analysis does not constitute a systematic review or meta-analysis. Furthermore, the core data indicators refer to different time periods; for instance, catastrophic health expenditure data (2006, 2017, and 2023); efficiency data (2020); National Health Accounts (2020); and heath equity indicators (2021)—rendering trend interpretation inherently challenging. Post-COVID-19 disruptions to the health system are also not explicitly examined due to the limited availability of robust evidence.

## 4 Conclusion

Mauritius has built something rare—a welfare state that demonstrably reaches its poorest citizens with quality healthcare, free at the point of use. The sustained improvement in the UHC Service Coverage Index over four decades and the strongly pro-poor distribution of public health benefits are genuine achievements in a world where such outcomes are far more often aspirational than actual.

Failing to take decisive action, these gains may risk erosion. Out-of-pocket spending is rising. Hospital inefficiency is consuming resources the system cannot afford to waste. The demographic transformation is already reshaping demand in ways that existing financing arrangements were not designed to absorb. The path forward is neither to abandon the commitment to universal free healthcare nor to defend the status quo uncritically. It is to strengthen the quality of public care to match its equity achievements, protect vulnerable populations from catastrophic financial risk, eliminate inefficiencies, and plan with genuine foresight for the pressures of an aging society. The Mauritius welfare state was built on the conviction that investing in people is the surest foundation for national prosperity. Health is rightly a strategic investment that delivers long term social and economic benefits. Continued investment is essential to enhance efficiency, equity, and financial protection, and to advance universal health coverage. Renewing that conviction—with the urgency the evidence demands—is the key health policy task for the next coming decades.

Mauritius' experience in advancing toward Universal Health Coverage offers valuable lessons that can meaningfully inform and inspire other Small Island Developing States.
